# Genotyping by Sequencing in Almond: SNP Discovery, Linkage Mapping, and Marker Design

**DOI:** 10.1534/g3.117.300376

**Published:** 2017-11-15

**Authors:** Shashi N. Goonetilleke, Timothy J. March, Michelle G. Wirthensohn, Pere Arús, Amanda R. Walker, Diane E. Mather

**Affiliations:** * School of Agriculture, Food and Wine, Waite Research Institute, The University of Adelaide, Glen Osmond, 5064, Australia; † Institut de Recerca i Tecnologia Agroalimentàries, Centre de Recerca en Agrigenòmica, Consejo Superior de Investigaciones Científicas–Institut de Recerca i Tecnologia Agroalimentàries–Universitat Autònoma de Barcelona–University of Barcelona, Campus Universitat Autònoma de Barcelona, 08913, Spain; ‡ Agriculture and Food, Waite Campus, Commonwealth Scientific and Industrial Research Organisation, Glen Osmond, 5064, Australia

**Keywords:** *Prunus dulcis*, single nucleotide polymorphisms, allele-specific molecular markers, composite linkage map, shell hardness

## Abstract

In crop plant genetics, linkage maps provide the basis for the mapping of loci that affect important traits and for the selection of markers to be applied in crop improvement. In outcrossing species such as almond (*Prunus dulcis* Mill. D. A. Webb), application of a double pseudotestcross mapping approach to the F_1_ progeny of a biparental cross leads to the construction of a linkage map for each parent. Here, we report on the application of genotyping by sequencing to discover and map single nucleotide polymorphisms in the almond cultivars “Nonpareil” and “Lauranne.” Allele-specific marker assays were developed for 309 tag pairs. Application of these assays to 231 Nonpareil × Lauranne F_1_ progeny provided robust linkage maps for each parent. Analysis of phenotypic data for shell hardness demonstrated the utility of these maps for quantitative trait locus mapping. Comparison of these maps to the peach genome assembly confirmed high synteny and collinearity between the peach and almond genomes. The marker assays were applied to progeny from several other Nonpareil crosses, providing the basis for a composite linkage map of Nonpareil. Applications of the assays to a panel of almond clones and a panel of rootstocks used for almond production demonstrated the broad applicability of the markers and provide subsets of markers that could be used to discriminate among accessions. The sequence-based linkage maps and single nucleotide polymorphism assays presented here could be useful resources for the genetic analysis and genetic improvement of almond.

Almond (*Prunus dulcis* Mill. D. A. Webb) is an important nut crop with an annual global production of 1.2 million tons (United States Department of Agriculture 2015). Almond breeding relies mostly on phenotypic assessment of parents, crossing between selected parents, vegetative propagation of progeny, and phenotypic selection among progeny, with only limited use of molecular information (Scorza 2001; Sánchez-Pérez *et al.* 2007; Gradziel 2009; Koepke *et al.* 2013). Development and implementation of modern molecular tools could support genetic mapping and the precision of the almond breeding process.

Almond is an outcrossing species with a gametophytic self-incompatibility system. Genetic mapping in almond has therefore used the pseudotestcross strategy (Arús *et al.* 1994; Viruel *et al.* 1995; Tavassolian *et al.* 2010; Font i Forcada *et al.* 2012, 2015; Fernández i Martí *et al.* 2013), which provides a linkage map for each parent. The first almond linkage maps to include all eight linkage groups were constructed based on application of isozyme and restriction fragment length polymorphism (RFLP) markers to progeny from a cross between the almond cultivars “Ferragnès” and “Tuono” (Viruel *et al.* 1995). Subsequently, a reference linkage map for almond was established with the application of isozyme and RFLP markers to progeny from a cross between the almond cultivar “Texas” and the peach (*P. persica* L. Batsch) cultivar “Earlygold” (Joobeur *et al.* 1998). Simple sequence repeat (SSR) markers were later added to this map (Aranzana *et al.* 2003; Dirlewanger *et al.* 2004). Other marker types that have been mapped in almond include random amplified polymorphic DNA (RAPD) markers, inter-SSR (ISSR) markers, sequence characterized amplified region markers, and single nucleotide polymorphism (SNP) markers (Joobeur *et al.* 2000; Wu *et al.* 2009, 2010; Tavassolian *et al.* 2010; Donoso *et al.* 2016). Among these, SNPs are particularly promising as they are the most abundant sequence differences in plants and they are usually biallelic and codominant. They have been used in many plant species, including almond (Wu *et al.* 2009, 2010; Donoso *et al.* 2016; Sorkheh *et al.* 2017).

With next-generation sequencing (NGS), it is possible to discover and directly assay large numbers of sequence polymorphisms without prior knowledge about the polymorphisms or their genomic positions. Given the size and complexity of plant genomes, NGS-based polymorphism discovery and genotyping benefit from the preparation of reduced representation libraries (Miller *et al.* 2007; Baird *et al.* 2008; Elshire *et al.* 2011; Peterson *et al.* 2012; Poland *et al.* 2012). Among various available library preparation protocols, the method proposed for genotyping by sequencing (GBS) by Elshire *et al.* (2011) is simple and makes it possible to discover thousands of SNPs. This method has been applied in many plant species, including peach (Bielenberg *et al.* 2015), sweet cherry (*P. avium* L.) (Guajardo *et al.* 2015), Japanese plum (*P. salicina* Lindl.) (Salazar *et al.* 2017), and apricot (*P. armeniaca* L.) (Gürcan *et al.* 2016).

While GBS can be a cost-effective approach for the initial discovery and mapping of large numbers of SNPs, the quality of the linkage maps produced is somewhat limited by genotyping errors and missing data. One way to address this limitation is to develop allele-specific assays for SNPs discovered by GBS and to apply these to the mapping population to obtain more accurate and complete data. Such assays may also be useful for application to materials beyond those that were included in the GBS library. Assays for individual SNPs are particularly useful when only one or a few markers are to be tested, as in marker-assisted selection for one or a few loci. Among the many technologies that can be used to assay SNPs, allele-specific Kompetitive Allele Specific PCR (KASP) assays (LGC Genomics, Teddington, United Kingdom) are now widely used in plant genetics and breeding (*e.g.*, Babiker *et al.* 2016; Rasheed *et al.* 2016; Tan *et al.* 2017).

In this research, the GBS protocol was adapted for almond and applied to F_1_ progeny from a cross between the almond cultivars “Nonpareil” and “Lauranne” to discover SNPs and construct linkage maps. KASP assays were then developed for a subset of the SNPs and were applied to the original mapping population and to additional materials. Quantitative trait loci (QTL) for shell hardness were mapped for Nonpareil and for Lauranne.

## Materials and Methods

### Plant materials and DNA samples

The almond clones used in this research were Nonpareil, Lauranne, “Chellaston,” “Constantí,” “Ferraduel,” “Glorieta,” “Johnston,” “Mandaline,” “Marta,” R1065, “Somerton,” “Tarraco,” “Vairo,” “White,” and 12-350 (Supplemental Material, Table S1 in File S1). In addition, 320 F_1_ progeny were used from crosses involving Nonpareil: 320 from Nonpareil × Lauranne (N × L), 349 from Nonpareil × Constantí (N × C), 207 from Nonpareil × Tarraco (N × T) and 198 Nonpareil × Vairo (N × V). Nonpareil is of particular interest because it is a major cultivar in both California and Australia.

Of the 320 N × L progeny, 89 had been previously used for genetic mapping by Tavassolian *et al.* (2010). For these clones and for Lauranne, the DNA samples used were residual samples from the earlier mapping work. These samples, which had been extracted from young leaves using the Lamboy and Alpha DNA extraction method (Lamboy 1998), were checked for DNA quality by electrophoresis on 1% agarose gels, quantified using PicoGreen intercalating dye (Invitrogen, Carlsbad, CA), and normalized to a working concentration of 20 ng/µl. For the other 231 N × L progeny, and for all of the other almond clones mentioned above, DNA was extracted from young leaves using an Oktopure DNA extraction protocol that had been optimized for almond (LGC Limited, Teddington).

In addition, some use was made of rootstock materials that are available in Australia: “Adafuel,” “Atlas,” “Bright’s Hybrid 1,” “Cornerstone,” “Felinem,” “Garnem,” “GF 557,” “Hansen 536,” “Krymsk 86,” “Monegro,” “Nemaguard,” “Nickels,” “Penta,” “Tetra,” and “Viking” (Table S2 in File S1). For these materials, DNA was extracted from young leaves using the method of Thomas and Scott (1993) followed by sodium chloride/ethanol precipitation.

### Library construction and sequencing

To select a restriction enzyme that might be suitable for digestion of the almond genome, *in silico* restriction of the peach whole genome sequence assembly v1.0 (www.rosaceae.org) was conducted using Biopython (Cock *et al.* 2009) for each of three methylation-sensitive restriction enzymes: *Ape*KI, *Pst*I, and *Hpa*II. The enzyme *Ape*KI, which has been used in GBS for other plants (Elshire *et al.* 2011; Lu *et al.* 2013; Bielenberg *et al.* 2015; Guajardo *et al.* 2015; Kujur *et al.* 2015; Gürcan *et al.* 2016; Salazar *et al.* 2017), was selected. Of the three enzymes, it was predicted to yield the highest number of fragments within the size range that is considered suitable for GBS (between 150 and 500 bp) (Table S3 in File S1). Further, it had been reported to generate uniform libraries with the degree of complexity reduction that is required for sequencing (He *et al.* 2014).

Barcode, primer, and adapter sequences for *Ape*KI (Table S4 in File S1) were obtained from http://www.maizegenetics.net/genotyping-by-sequencing-gbs. Barcodes, adapters, and primers were synthesized by Sigma Aldrich (Castle Hill, Australia). The complementary top and bottom strands of each barcode and adapter were diluted to 10 µM with 10× adapter buffer and annealed using the following PCR conditions: 95° for 1 min, followed by ramping down to 30° by 1° per cycle. The resulting double-stranded barcode and adapters were diluted separately in 1× TE to 0.6 ng/µl, quantified using PicoGreen intercalating dye, and normalized to 0.1 µM with 1× TE. Each barcode solution was mixed with the adapter solution in a 1:1 ratio in one well of a 96-well plate.

To select an appropriate ratio between adapter and DNA concentrations, a titration experiment was carried out. For this, a pooled DNA sample was prepared by mixing equal amounts of DNA from 10 N × L F_1_ progeny. Eight 200-ng samples of DNA were drawn from this pooled sample. After addition of 3.2 U of *Ape*KI in 2 µl 10× NEB buffer (New England Biolabs, Ipswich, MA) and water to bring the final volume to 20 µl, these samples were incubated for 2 hr at 75°. One of eight quantities of adapter (2, 5, 8, 10, 12, 15, 18, or 20 µl of a 0.1 M adapter solution), 10 µl of a solution containing 200 U of T4 DNA ligase (New England Biolabs), and 5 µl of 10× ligation buffer were added. Samples were incubated at 22° for 2 hr and then at 65° for 20 min. Ligation products were purified using a PureLink PCR Purification Kit (Invitrogen) as per the manufacturer’s instructions. Each purified ligation product was resuspended in a final volume of 50 µl. For the final library, 10 µl of each purified ligation product was used in a 25-µl PCR reaction with 2 µl of the 10-µM paired-end primers 5′-AATGATACGGCGACCACCGAGATCTACACTCTTTCCCTACACGACGCTCTTCCGATCT-3′ and 5′-CAAGCAGAAGACGGCATACGAGATCGGTCTCGGCATTCCTGCTGAACCGCTCTTCCGATCT-3′ along with 12.5 µl of Taq 2X Master Mix (New England Biolabs). The PCR conditions used were as follows: 30 sec at 95°, 15 cycles of 30 sec at 95°, 20 sec at 65°, 30 sec at 68°, followed by a final extension at 72° for 5 min. Each amplified library was purified as described above and eluted in a final volume of 30 µl. Each library (2 µl) was run on 2% agarose at 90 V for 30 min to evaluate the library and the adapter dimer peaks. An adapter concentration of 4.5 ng in a volume of 15 µl was selected because it provided a satisfactory library with no adapter dimer peak.

Library preparation was carried out using 200 ng (10 µl of 20 ng/µl) of DNA from each of the initial 89 N × L progeny and each of three aliquots of DNA from Nonpareil and Lauranne. The same procedure was carried out for a water sample as a negative control. Initial reactions were carried out in a 96-well plate using a separate well for each sample. After adapter ligation, samples were pooled for purification, PCR amplification, evaluation, and sequencing. The pooled library was sequenced using single-end sequencing (100-bp reads) on one flow-cell lane of an Illumina HiSeq 2000 instrument at the Australian Genome Research Facility (Melbourne, Australia).

### SNP discovery

The GBS sequence data were analyzed using the Universal Network Enabled Analysis Kit (UNEAK) pipeline in TASSEL 3.0 software (Bradbury *et al.* 2007; Lu *et al.* 2013). This pipeline permits SNP calling based solely on GBS tag sequence data, without requiring a reference genome sequence. The output was filtered to select SNPs with at least 80% coverage across samples, a minimum read depth of 5, and a minimum relative heterozygosity value (ratio of heterozygotes to homozygotes) of 0.01. The level of almond genome coverage obtained was estimated using the Lander–Waterman equation (Lander and Waterman 1988).

### Construction of linkage maps

For the construction of initial framework linkage maps for Nonpareil and Lauranne, tag pairs with minor allele frequencies (MAF) between 0.2 and 0.3 in the mapping population were selected. This was based on the expectation of a MAF of 0.25 for the most informative SNPs (those that are heterozygous in one parent and homozygous in the other parent). The resulting data set was separated into two parental data sets based on whether the tags were homozygous in Nonpareil and heterozygous in Lauranne, or vice versa. Each of these data sets was further filtered to retain only the SNPs missing no more than 20 data points per marker and with segregation ratios not deviating significantly from 3:1 (α = 0.05). Separate parental linkage maps were constructed for Nonpareil and Lauranne using a double pseudotestcross strategy implemented using the backcross (BC) format in ASMap R package version 1.0-1 (Taylor and Butler 2017) with the following map construction strategy:

1. An initial framework linkage map was constructed using data from progeny for which there were no missing data for the selected SNPs. Data for a few SSR, RAPD, and ISSR markers that Tavassolian *et al.* (2010) had reported to be homozygous in one parent and heterozygous in the other parent were included in addition to data for the selected SNPs. Linkage mapping was carried out using the minimum spanning tree map algorithm (MSTmap) (Wu *et al.* 2008) as implemented in ASMap to assign markers to linkage groups and to order them within linkage groups. A *P*-value of 0.0001 was used to declare whether markers belong to the same linkage group. The Kosambi mapping function (Kosambi 1944) was used to calculate genetic distances in cM.
2. For each linkage group, ASMap was used to generate a heat map (rf/LOD plots) to evaluate pairwise associations between markers. For cases in which markers appeared to have had their alleles assigned to the incorrect parents, genotype designations were reassigned using the “switchAlleles” function of the R/qtl R package version 1.41-6 (Broman *et al.* 2003). Maps were then reestimated using the mstmap.cross function.
3. To further improve the quality of the Nonpareil and Lauranne linkage maps, markers were checked for segregation distortion and numbers of double crossover events involving adjacent marker intervals. Markers were removed if their segregation ratio deviated significantly from 1:1 (α = 0.05) and/or if they were associated with high numbers of apparent double crossover events. Maps were then reestimated using the mstmap.cross function.
4. The orientation of each linkage group of the Nonpareil and Lauranne maps was established by comparing the maps constructed using the SNP data with the published maps of Tavassolian *et al.* (2010). This was done using the AlignCross function of ASMap.

From the resulting framework maps for Nonpareil and Lauranne, a set of GBS markers from the eight linkage groups was selected for the design of allele-specific assays, with the objective of obtaining markers spaced at ∼10-cM intervals throughout the genome.

### Primer design

Primer sets were designed for SNPs that had been discovered and mapped based on the GBS data. Some of these were heterozygous in Nonpareil and homozygous in Lauranne, and others were heterozygous in Lauranne and homozygous in Nonpareil. For some of these, it was possible to design primers based solely on the GBS data. For others, the SNPs were too close to one end of the GBS tags. For these, the tags were aligned to Nonpareil genomic contig sequences using the BLAST tool in Geneious software version 9.1.3 (Kearse *et al.* 2012) to obtain sequences of ∼100 bp with the SNPs located near their midpoints. Each SNP-bearing sequence was used to design a set of three primers (two allele-specific primers and one common primer) using Kraken software (LGC Limited). The primer sets were named using the prefix WriPdK, with Wri referring to the Waite Research Institute, Pd referring to *P. dulcis*, and K referring to KASP technology.

### Application of KASP assays

A total of 309 primer sets (146 designed for SNPs that were heterozygous in Nonpareil and 162 designed for SNPs that were heterozygous in Lauranne) were assayed on a panel consisting of DNA samples of Nonpareil, Lauranne, and seven N × L F_1_ progeny, with a water sample included as a negative control. Samples of 10 ng of DNA (5 µl of 2 ng/µl) were dried at 55° for 1 hr. Aliquots of a primer mixture (0.028 µl, containing 12 µM of the allele-specific primers and 30 µM of the common primer) and of 1× KASP Master Mix (1.972 µl; LGC Limited) were added to each reaction sample. PCR amplification was conducted using the standard KASP PCR protocol in a Hydrocycler-^16^ PCR system (LGC Limited). Fluorescence was detected in a Pherastar Plus plate reader (BMG LABTECH, Ortenberg, Germany). Data were analyzed using Kraken software (LGC Limited). Primer sets that detected polymorphism in the validation panel were selected and assayed on 311 N × L progeny: 80 of the 89 progeny that had been used to prepare the GBS library, plus 231 others. The same primer sets were also assayed on the panel of almond clones. Genotypic calls were compared among clones using Flapjack software version 1.16 (Milne *et al.* 2010). Selected markers (those that were heterozygous in one parent and homozygous in the other) were assayed on the N × C, N × T, and/or N × V progeny.

### Linkage mapping using KASP markers

Linkage maps were constructed for each parent using KASP marker data from 80 N × L progeny, 231 N × L progeny, 349 N × C progeny, 207 N × T progeny, and 198 N × V progeny, using the procedures described for the construction of the initial framework linkage map. Maps were drawn using MapChart version 2.3 software (Voorrips 2002).

Data from 985 progeny from four crosses (N × C, N × L, N × T, and N × V) were used to construct a composite map for Nonpareil. This was achieved using a pseudotestcross mapping strategy with data coded in the BC format, considering only those markers for which Nonpareil was heterozygous. In populations for which the other parent was homozygous at a marker, the codes “ab” and “aa” were assigned to heterozygous and homozygous progeny, respectively. In populations for which the other parent was also heterozygous, all progeny were coded as having missing data.

Recombination fractions between adjacent markers were estimated using the R/qtl package version 1.41-6 (Broman *et al.* 2003). Recombination fractions were converted to map distances using the Kosambi mapping function (Kosambi 1944). The resulting composite map was compared with Nonpareil maps that had been constructed using data from individual populations. Markers for which there were substantial inconsistencies among maps were removed and the mapping analysis was repeated. Markers that had been mapped in only one population were assigned positions in the composite map based on their positions relative to flanking markers. The final composite map was drawn using MapChart version 2.3 software (Voorrips 2002).

### Comparative mapping between almond and peach

Each unique GBS sequence read obtained for Nonpareil and Lauranne that was at least 64-bp long and had sequence coverage ≥10 was aligned against the peach (*P. persica*) whole genome sequence assembly v2.0.a1 (www.rosaceae.org) using the BLAST+ tool version 2.2.27 (http://www.ncbi.nlm.nih.gov/blast). Each sequence read was considered to have been anchored to the peach genome if it mapped to a unique site with >90% sequence similarity and an *E*-value <1*e*^−15^. For sequences that met these criteria and for which marker assays had been developed, the Circlize R package version 0.4.1 (Gu *et al.* 2014) was used to compare the genetic positions in almond with physical positions in the eight main scaffolds of the peach genome assembly.

### QTL mapping for shell hardness

Shell hardness was evaluated in 2015 for 180 N × L progeny. For each tree, a random sample of 10 nuts was weighed to obtain in-shell weight. The nuts were then cracked open using a nutcracker, and kernels were weighed. The shell-hardness percentage was calculated as suggested by Rugini (1986): (kernel weight/in-shell weight) × 100%. According to this measure, almond nuts may be classified as paper shell (≥55%), soft shell (45–54%), semihard shell (35–45%), hard shell (25–34%), or stone shell (≤24%). QTL for this trait were mapped using the R/qtl package version 1.41-6 (Broman *et al.* 2003), with the function Scanone used to test for putative QTL at 1-cM intervals throughout the genome. Significance was declared by comparing LOD values to a threshold determined using 10,000 permutations and a genome-wide significance level of 0.05.

### Polymorphism detection in rootstocks

Using the KASP assay procedures described above, 253 SNPs (128 heterozygous in Nonpareil and 125 heterozygous in Lauranne) were assayed on duplicate samples of DNA samples extracted from the rootstock accessions.

### Data availability

Information on the parentage and origin of the almond clones and rootstocks used in this research is in Tables S1 and S2 in File S1. Sequence data for 89 N × L progeny have been deposited in the National Center for Biotechnology Information Short Read Archive: study SRR5722967. Information on fragment size distributions from *in silico* digestion of the peach genome sequence is in Table S3 in File S1. Barcode and primer sequences used for GBS are in Table S4 in File S1. Contig sequences for Nonpareil have been deposited in the European Nucleotide Archive under accession number PRJEB23106. Primer sequences for KASP assays are in Table S5 in File S1. Linkage maps for Nonpareil are in Table S6 in File S1. Linkage maps for Lauranne, Constantí, Tarraco, and Vairo are in Table S7 in File S1. Results obtained from the application of KASP markers to almond clones and rootstocks are in Tables S8 and S11 in File S1, respectively. The best BLAST hits in the peach genome for SNP-bearing tags from Nonpareil and Lauranne are in Tables S9 and S10 in File S1, respectively. The genotypic and phenotypic data used for QTL analysis are in Tables S12 and S13 in File S1.

## Results

### Sequence data

**Figure 1:**
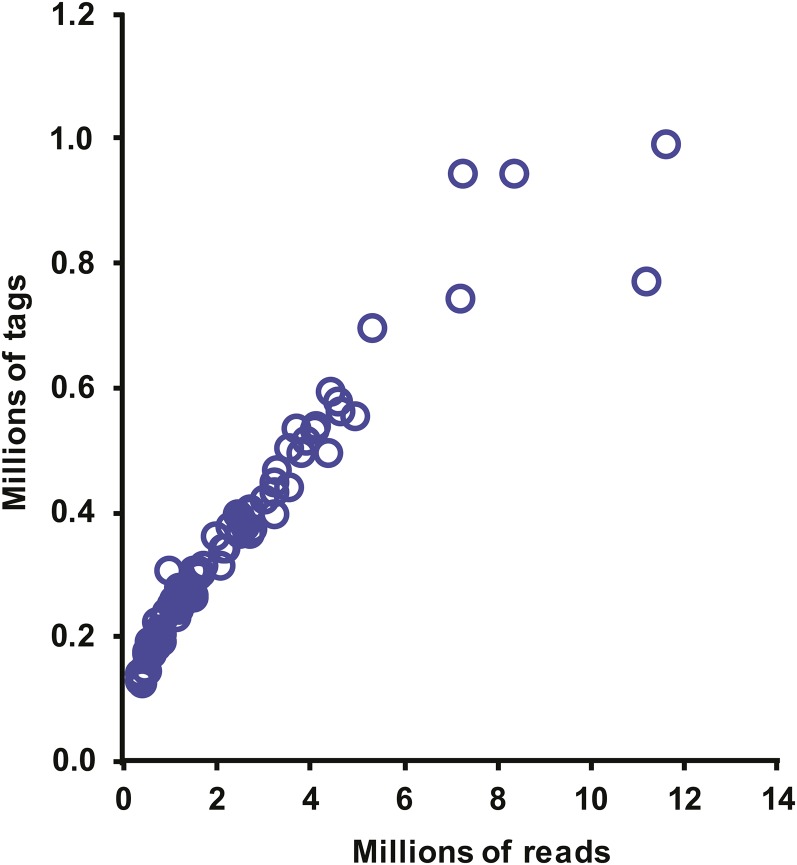
Number of unique sequence tags *vs.* number of sequence reads. Relationship between the number of sequence reads obtained and the number of unique 64-bp sequence tags obtained from GBS of 89 N × L F_1_ progeny.

The GBS library generated 21.6 Gb of sequence data, with a total of 186 million sequence reads (a mean of 2.1 million per sample). Linear regression analysis indicated a strong positive relationship between the number of sequence reads and the number of tags for each individual (*R*^2^ = 0.92, *P* < 0.0001, Figure 1).

**Figure 2:**
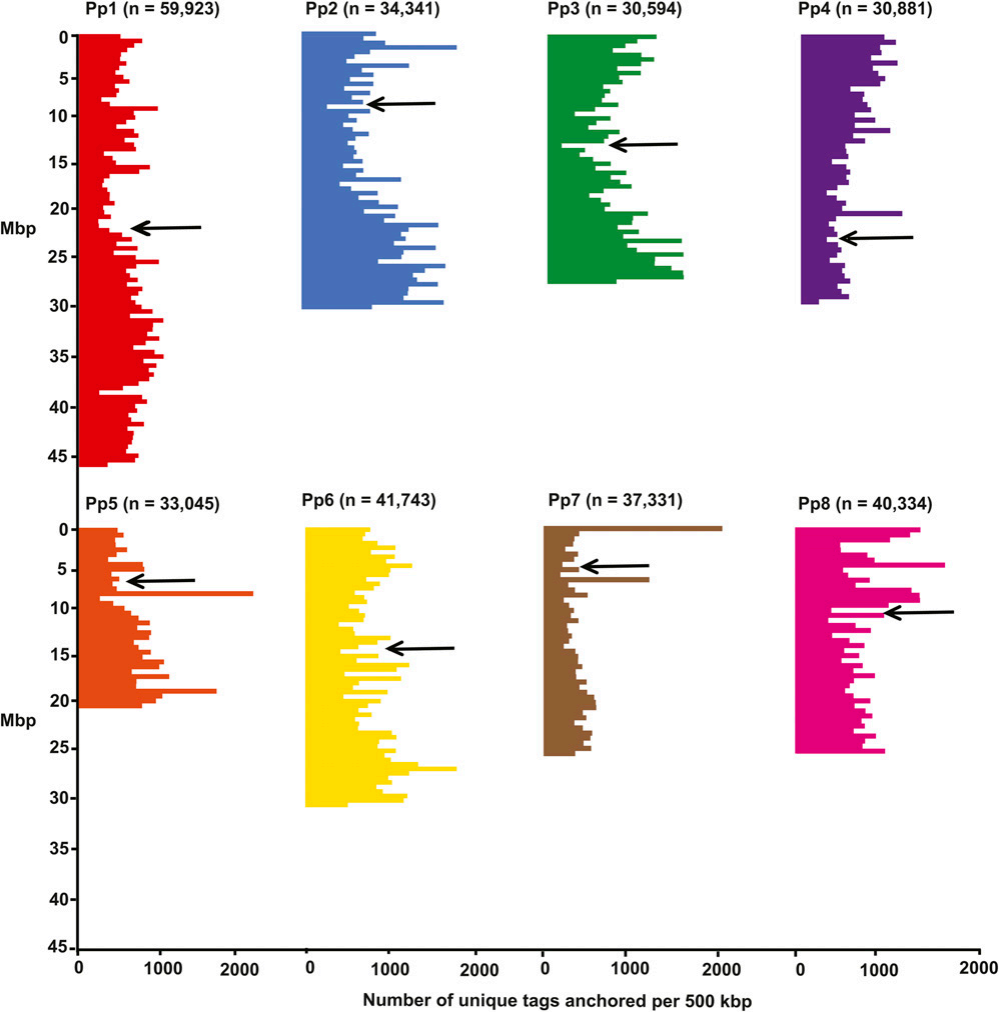
Anchoring of almond sequence tags to peach genome. Numbers of unique tags anchored to each 500-kbp region of each of the eight main scaffolds (Pp1–Pp8) of the peach whole genome sequence assembly v2.01a1. For each scaffold, the total number of unique tags is given in parentheses and the estimated position of the centromere is indicated with an arrow.

Across all samples, a total of 453,648 unique tags was obtained. Of these tags, 308,971 (68%) were anchored to the peach genome, with between 30,594 and 59,923 mapping to each of the eight main scaffolds (Pp1–Pp8) (Figure 2) and 6088 mapping to other scaffolds. Tags were mapped throughout the entire length of each main scaffold, but with some variation in the marker density. There were a few regions (*e.g.*, on Pp5 and Pp7) with very high density.

From the unique tags, 11,936 SNP-containing tag pairs were identified. With the application of a series of filters, >300 tag pairs that were considered suitable for mapping were selected for each of Nonpareil and Lauranne.

### Linkage maps for Nonpareil and Lauranne

An initial framework map constructed for Nonpareil based on complete data for 52 progeny had 327 markers (310 GBS, 9 SSR, 5 ISSR, and 3 RAPD) on eight linkage groups with a total length of 1152 cM (Table S6 in File S1). The initial framework map constructed for Lauranne was based on complete data from 55 progeny. It has eight linkage groups, 295 markers (279 GBS, 5 SSR, 8 ISSR, and 3 RAPD), and is 1371 cM long (Table S7 in File S1).

**Figure 3:**
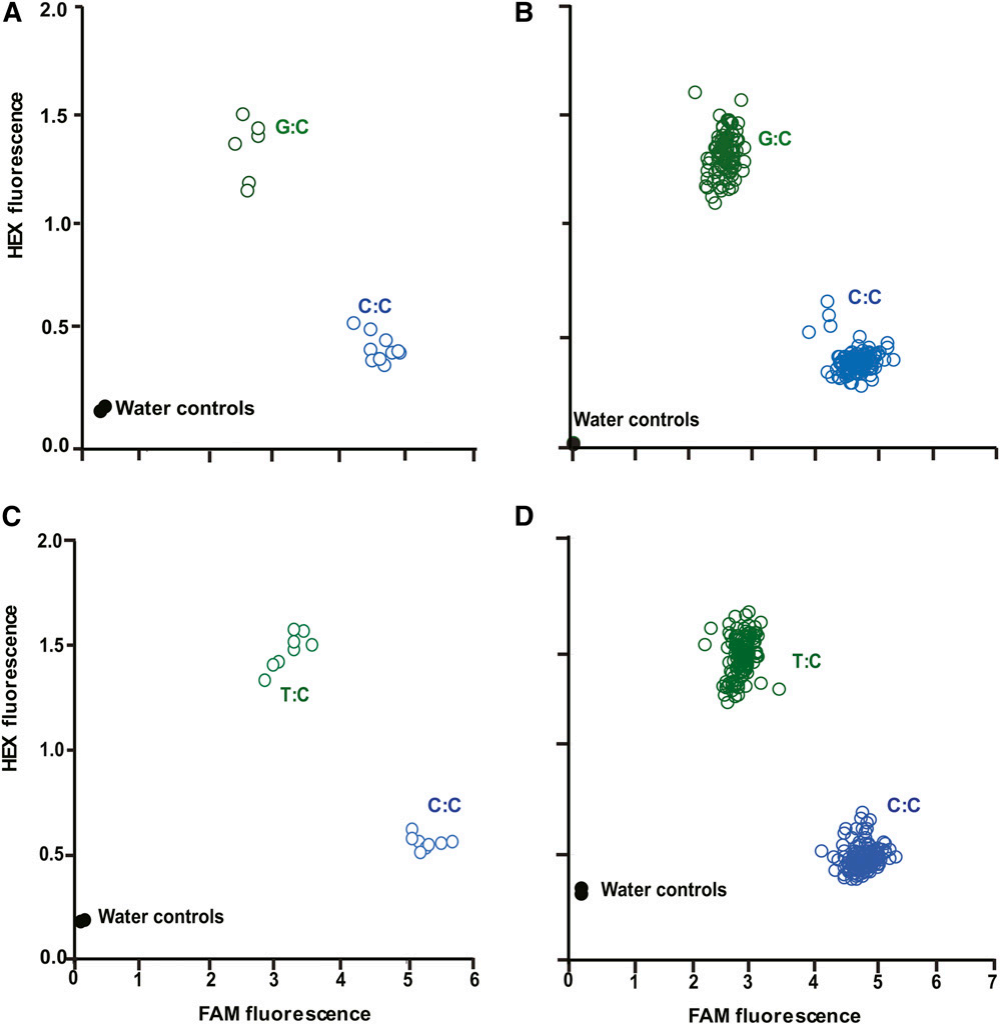
Examples of KASP assay results. Intensities of FAM and HEX fluorescence detected when two KASP assays (WriPdK7 and WriPdK69) were applied to Nonpareil, Lauranne, and N × L progeny. The WriPdK7 primers were designed for a SNP that is heterozygous (G:C) in Nonpareil and homozygous (C:C) in Lauranne. The WriPdK69 primers were designed for a SNP that is homozygous (C:C) in Nonpareil and heterozygous (T:C) in Lauranne. (A) WriPdK7 applied to Nonpareil (in duplicate), Lauranne (in duplicate), and seven N × L F_1_ progeny. (B) WriPdK7 applied to 231 N × L F_1_ progeny. (C) WriPdK69 applied to Nonpareil (in duplicate), Lauranne (in duplicate), and seven N × L F_1_ progeny. (D) WriPdK69 applied to 231 N × L F_1_ progeny.

Of the 149 KASP primer sets designed based on sequence tags that exhibited heterozygosity in Nonpareil (*e.g.*, Figure 3A), 138 detected polymorphism among the progeny (*e.g.*, Figure 3B). Of the 162 primer sets designed based on sequence tags that exhibited heterozygosity in Lauranne (*e.g.*, Figure 3C), 155 detected polymorphism among the progeny (*e.g.*, Figure 3D). None of the genotypic ratios observed for these polymorphisms deviated significantly from the expected 1:1 ratio.

**Figure 4:**
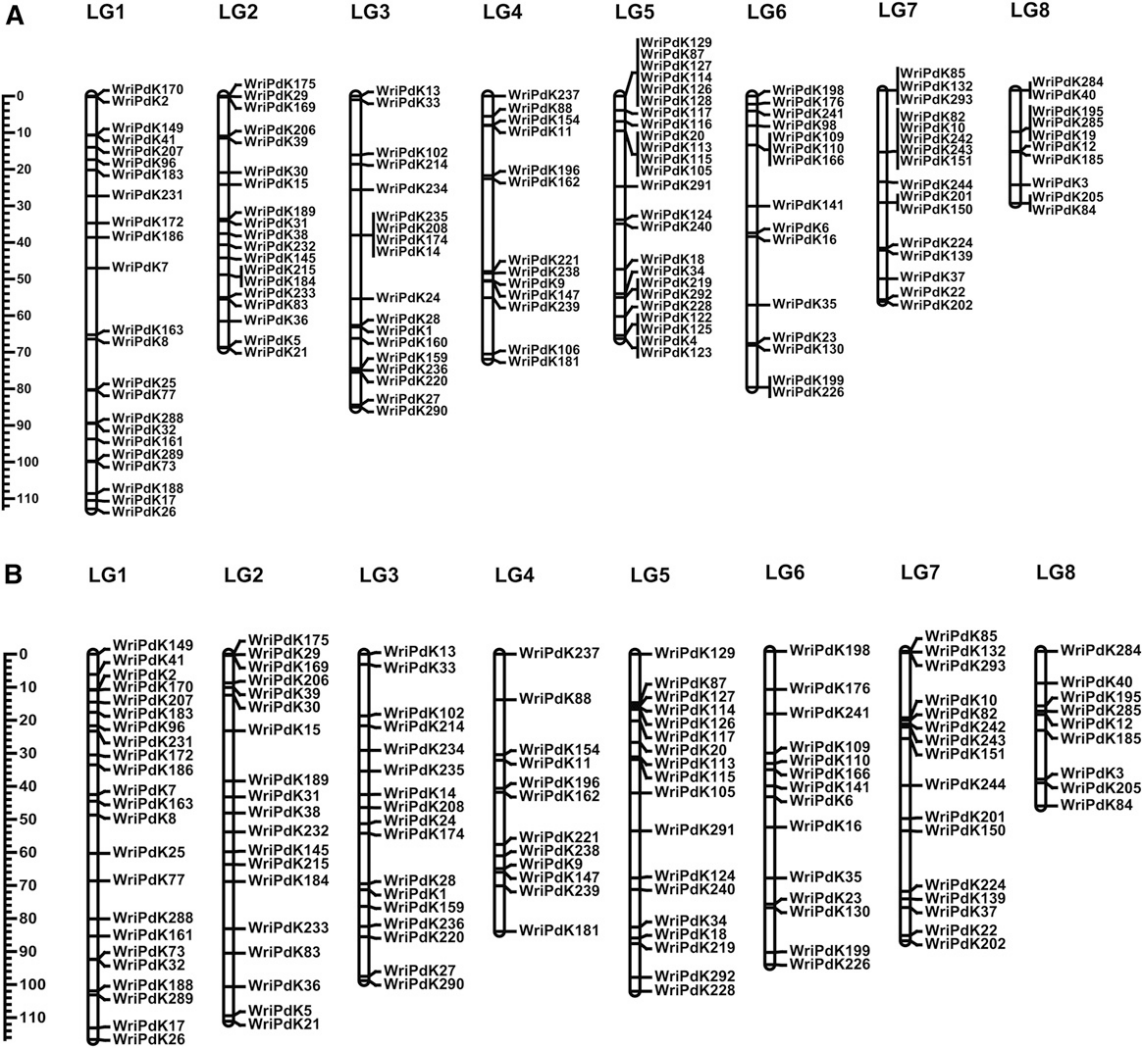
Nonpareil linkage maps. Linkage maps constructed for Nonpareil using genotypic data from KASP assays applied to (A) 231 N × L F_1_ progeny and (B) 985 F_1_ progeny from four crosses (N × L, N × C, N × T, and N × V).

**Figure 5:**
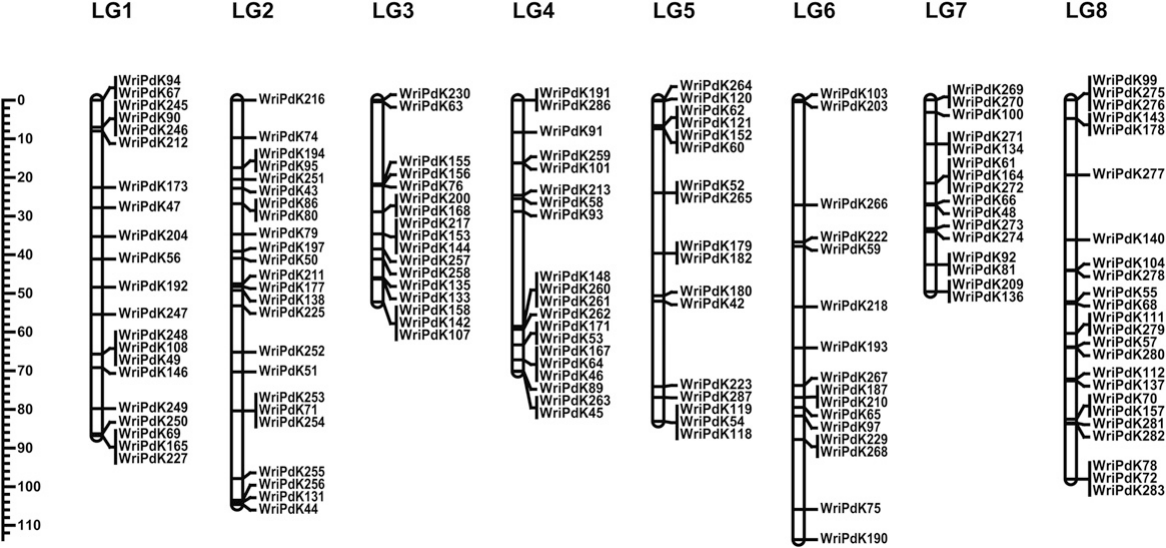
Lauranne linkage map. A linkage map constructed for Lauranne using genotypic data from KASP assays applied to 231 N × L F_1_ progeny.

Relative to the initial framework maps that were derived from GBS data, the linkage maps constructed based on KASP marker data for 231 N × L progeny had very similar marker orders, but were much shorter (Tables S6 and S7 in File S1). The KASP map for Nonpareil had 138 markers and a total length of 609 cM (Figure 4A). The KASP map for Lauranne had 155 markers and a total length of 659 cm (Figure 5).

### Polymorphisms among almond clones

**Figure 6:**
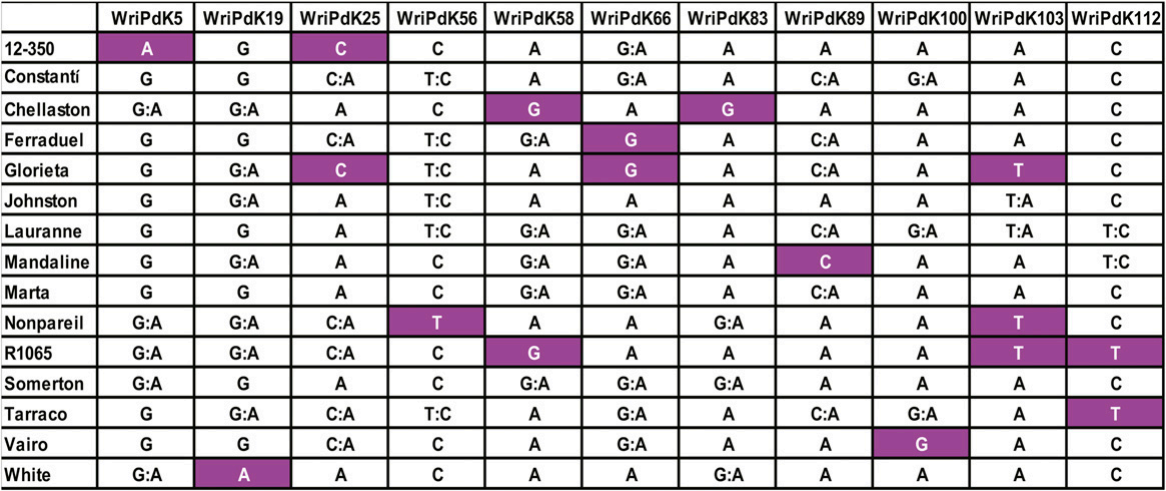
Genotypes of 15 almond clones for 11 KASP markers selected based on their ability to discriminate among these clones. For each marker, the least common genotype is shown in white text on a dark background.

Of the 261 KASP assays tested, 239 exhibited polymorphism among 14 almond clones other than Nonpareil and Lauranne (Table S9 in File S1). Of these markers, 111 had been designed based on heterozygosity in Nonpareil, and 128 based on heterozygosity in Lauranne. Among the 14 other almond clones on which these markers were assayed, all except Marta and Somerton could be distinguished from all others by just one marker. A total of 11 KASP assays were selected (Figure 6) that, in combination, could be useful for distinguishing among all of the clones that were examined here.

### Linkage maps based on N × C, N × T, and N × V

Of the 138 KASP markers that were developed based on Nonpareil heterozygosity and mapped using N × L progeny, 92, 85, and 103 markers detected polymorphism in N × C, N × T, and N × V, respectively. Of the 155 KASP markers that were derived based on Lauranne heterozygosity and mapped using N × L progeny, 68, 40, and 56 markers detected polymorphism in N × C, N × T, and N × V, respectively. In addition, several markers that had not exhibited polymorphism among N × L progeny were found to be polymorphic in other populations. Linkage maps for Nonpareil that were based on N × C, N × T, and N × V had 90, 82, and 94 markers, respectively, with total lengths of 439, 569, and 553 cM, respectively (Table S6 in File S1), and with marker orders very similar to those obtained with the N × L population. Linkage maps developed for Constantí, Tarraco, and Vairo had 65, 39, and 52 markers, respectively, with total lengths of 382, 295, and 148 cM (Table S7 in File S1).

### QTL for shell hardness

**Figure 7:**
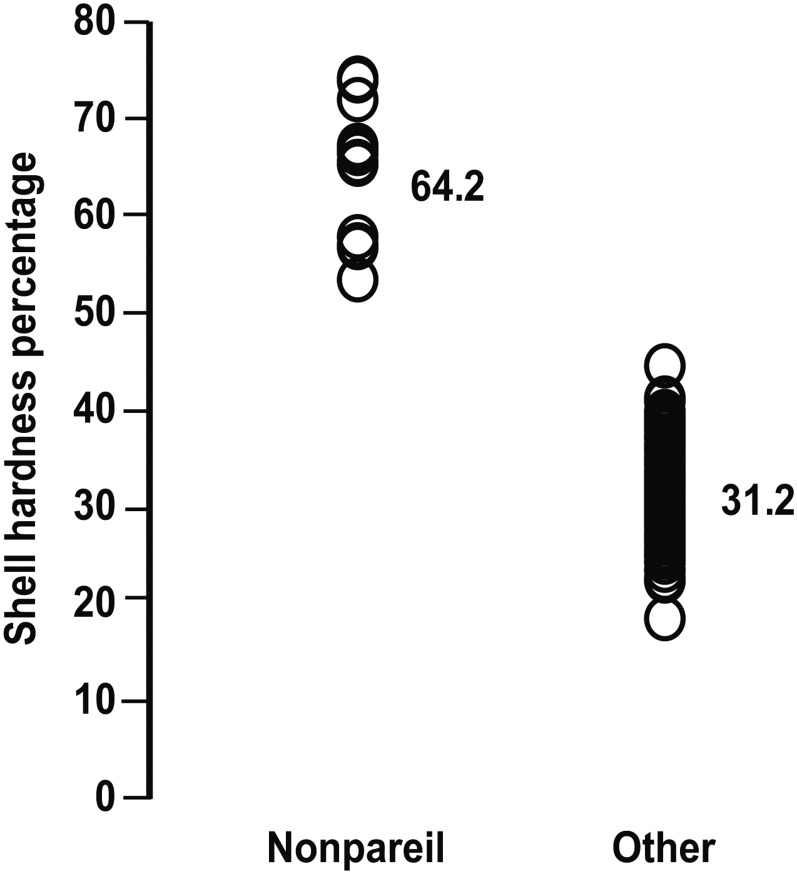
Marker-based discrimination of progeny with the paper-shell trait. Shell-hardness percentages and means for two sets of N × L F_1_ progeny, one selected to have the Nonpareil genotypic combination across six markers (WriPdK251 and WPdK50 on LG2; WriPdK129, WriPdK18, and WriPdK264 on LG5; WriPdK282 on LG8) are associated with shell hardness and the other consisting of progeny with other genotypic combinations at those markers.

For Nonpareil, QTL for shell hardness were detected in two regions, both on linkage group 5 (LG5). For Lauranne, QTL for shell hardness were detected in four regions: one on LG2, two on LG5, and one on LG8 (Table 1). In all of these regions, Nonpareil-like genotypes were associated with softer shells (higher mean shell hardness). Of the 180 progeny that were evaluated for shell hardness, just 17 had the Nonpareil-like genotype in all QTL regions. Like Nonpareil, these progeny exhibited the “paper-shell” trait (very high shell-hardness percentage). The genotypes at six markers (WriPdK251 and WPdK50 on LG2; WriPdK129, WriPdK18, and WriPdK264 on LG5; and WriPdK282 on LG8) were sufficient to separate progeny with the paper-shell trait from those with harder shells (Figure 7).

**Table 1:** QTL detected for shell-hardness percentage in Nonpareil and Lauranne based on evaluation of nuts harvested in 2015 from 180 N × L F_1_ progeny

Linkage Map	Linkage Group	Position (cM)	LOD	*R*^2^*^a^*
Nonpareil KASP map	LG5	0	2.5	9
		45	2.5	9
Lauranne KASP map	LG2	26	3.4	9
	LG5	0	4.2	11
		43	3.2	9
	LG8	87	3.4	9

a Percentage of phenotypic variance explained by the QTL.

### Composite linkage map for Nonpareil

The composite linkage map constructed based on data from the progeny of four Nonpareil crosses had 129 KASP markers with a total length of 741 cM (Figure 4B and Table S6 in File S1). Some markers that had collocated in Nonpareil genetic maps constructed for individual Nonpareil populations (N× L, N × C, N × T, and/or N × V) were separated in the composite map.

### Comparison of almond genetic maps with the peach genome

**Figure 8:**
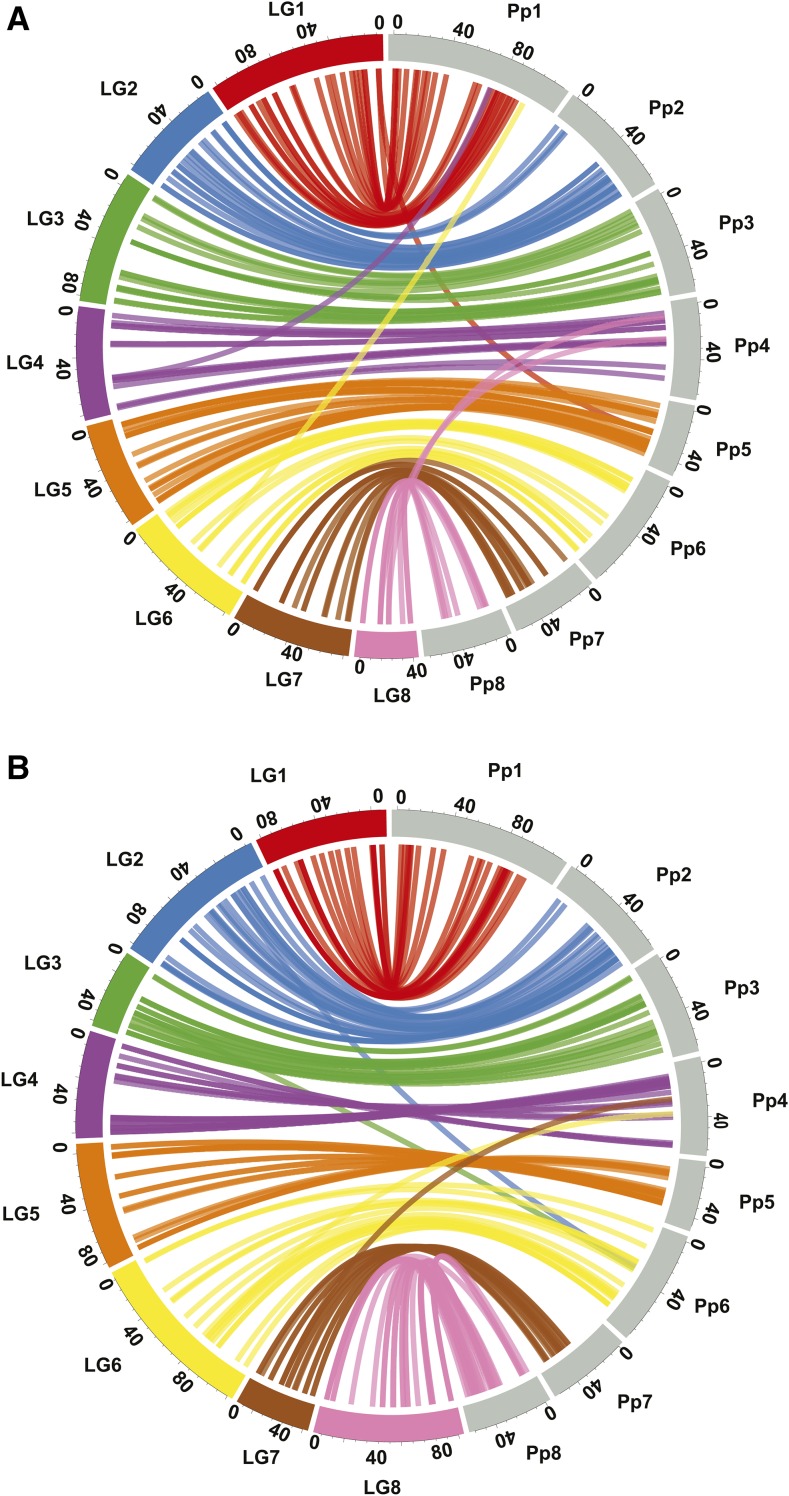
Comparisons of almond genetic maps with the peach genome sequence. (A) Nonpareil linkage groups 1–8 compared with peach scaffolds Pp1–Pp8. (B) Lauranne linkage groups 1–8 compared with peach scaffolds Pp1–Pp8. On the almond linkage groups, genetic distances are given in cM. On the peach scaffolds, physical distances are given in Mbp. Links between linkage groups and scaffolds indicate the positions at which markers genetically mapped in almond anchor to the genomic sequence of peach.

Comparison of marker positions on Nonpareil and Lauranne parental maps with positions on peach genome scaffolds confirmed the expected high synteny and collinearity between the almond and peach genomes (Figure 8 and Tables S9 and S10 in File S1). Almost all markers anchored to the expected peach scaffolds. For the Nonpareil map, the exceptions are a few markers that genetically mapped on LG1, LG4, LG6, and LG8 but anchored to peach scaffolds Pp5, Pp1, Pp1, and Pp4, respectively. For the Lauranne map, there were markers that genetically mapped on LG2, LG3, and LG6 but anchored to peach scaffolds Pp6, Pp6, and Pp4, respectively. There are also a few discrepancies in marker order between the almond genetic maps and the peach scaffolds (*e.g.*, at each end of Nonpareil LG4 and peach scaffold Pp4). A few areas of the peach genome are not well represented on one or both almond linkage maps. For example, only two markers from the Nonpareil LG7 map and no markers from the Lauranne LG7 map anchored between 0 and 7 Mbp on peach Pp7 scaffold. There are also some regions in which markers that are closely linked in almond (*e.g.*, at 27 cM on Nonpareil LG2 and at 20 cM on Lauranne LG8) anchored to physically distant positions on peach scaffolds.

### Polymorphisms among rootstocks

**Figure 9:**
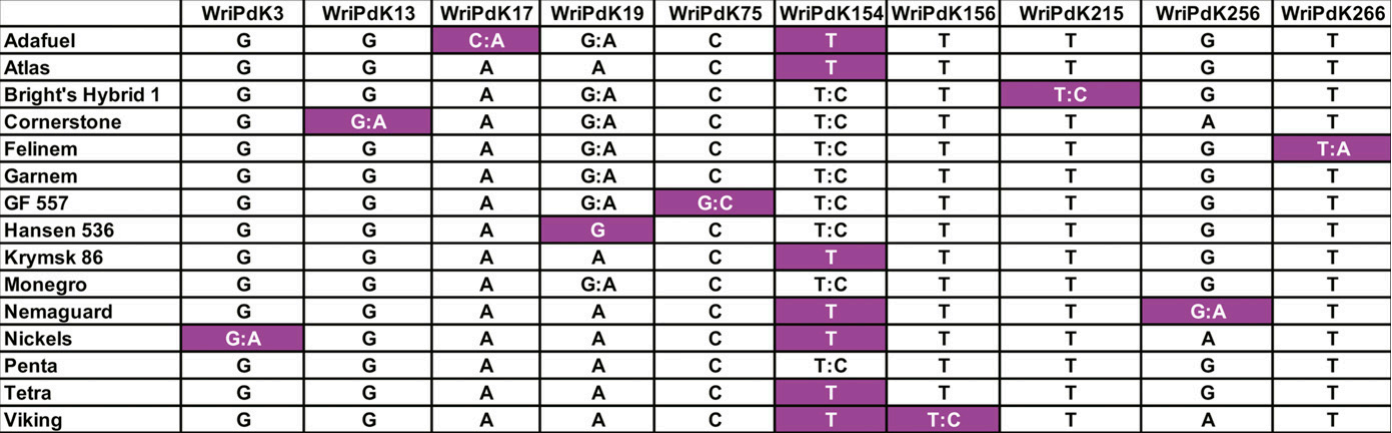
Genotypes of 15 rootstocks for 10 KASP markers selected based on their ability to discriminate among these rootstocks. For each marker, the least common genotype is shown in white text on a dark background.

Of the 220 KASP assays tested on rootstock materials, 169 were polymorphic and 66 were monomorphic (Table S11 in File S1). Of the 169 polymorphic assays, 93 had been designed based on Nonpareil heterozygosity, and 76 based on Lauranne heterozygosity. In most cases, just one marker was sufficient to distinguish a particular rootstock from all others. Penta and Tetra, both of which are derived from European plum, were very similar but there were four markers that distinguished between them. A total of 10 KASP assays were selected (Figure 9) that, in combination, could be useful for distinguishing among all of the rootstock materials that were examined here.

## Discussion

In this research, implementation of a GBS protocol enabled discovery of thousands of SNP-bearing GBS tags, providing an easy method to discover and assay SNPs without any prior sequence information. The restriction enzyme used here, *Ape*KI, is a type-II endonuclease that recognizes a degenerate 5-bp sequence (GCWGC, where W is A or T). It is useful for the reduction of sequence complexity, because it has relatively few recognition sites in the major classes of plant retrotransposons and will not cut if the 3′ base of the recognition sequence on the bottom strand is 5′ methyl cytosine (Söllner *et al.* 2006). It creates a 5′ overhang of 3 bp, providing sites for attachment of adapters to which primers can anneal to provide a uniform library for sequencing (He *et al.* 2014).

The mean number of sequence reads per sample that was obtained here (2.1 million) is similar to what has been reported for other *Prunus* species: 1.8 million for sweet cherry (Guajardo *et al.* 2015), 2.4 million for peach (Bielenberg *et al.* 2015), 2.3 million for Japanese plum (Salazar *et al.* 2017), and 3.5 million for apricot (Gürcan *et al.* 2016). Of the sequences generated, 68% were anchored to unique positions in the peach genome sequence assembly. This is higher than was reported for apricot (43%, Salazar *et al.* 2017), which is not as closely related to peach. Within a total of 224 Mb of the peach genome to which almond tags were anchored, the density of almond–peach SNPs was ∼1 per 22 kb. Similar results have been reported for sweet cherry (Guajardo *et al.* 2015). Some variation was observed in the numbers of sequence tags and SNPs mapped to each scaffold and in the anchor positions of tags within scaffolds. Possible reasons for this variation could include: (1) variation in the distribution of *Ape*KI restriction sites across the almond genome, (2) variation in DNA methylation across the almond genome, and (3) structural differences between the almond and peach genomes. The unusually high numbers of sequence tags obtained in some regions (*e.g.*, one at 6.5 Mbp on Pp5 and one at 0.5 Mbp on Pp7) may indicate that these regions are more polymorphic or more repetitive in almond than in peach. Consistent with this, one of these regions (at 6.5 Mbp on Pp5) corresponds with a region of LG5 in which many polymorphisms were genetically mapped for Nonpareil (albeit not for Lauranne). However, another one (at 0.5 Mbp on Pp7) is on a chromosome arm for which only two polymorphisms were mapped in Nonpareil and none were mapped for Lauranne. Lack of polymorphism in this region may not be limited to these materials, nor even to almond, as similar observations have been reported for this region for the sweet cherry cultivars Riverdale and Rainer (Guajardo *et al.* 2015). The consistent lack of polymorphism in certain genomic regions could reflect fixation of favorable alleles due to selection.

While the total number of high-quality SNPs obtained for almond (11,936) was sufficient for genetic mapping and higher than was obtained for sweet cherry (8476; Guajardo *et al.* 2015) it is lower than what was obtained for apricot (18,322; Gürcan *et al.* 2016) or plum (42,909; Salazar *et al.* 2017). One approach to increase the number of SNPs discovered would be to use less-stringent filters in the sequence analysis. The TASSEL GBS 3.0 SNP-calling pipeline, which was developed mainly for highly homozygous materials, is considered to be sensitive to low sequence depth in highly heterozygous materials (Hyma *et al.* 2015). Therefore, a stringent read depth cutoff value of five was applied per marker call. With a value of three, a larger number of GBS tags (>600) could have been selected for mapping, but maps constructed on this basis (data not shown) had very long linkage groups (>300 cM); some of the additional SNPs may have been spurious.

Another approach to increase the number of SNPs discovered could be to increase sequence depth. In this analysis, there was a strong positive relationship (*R*^2^ = 0.92) observed between total read number and the total number of unique tags, indicating that additional unique tags and SNPs might have been discovered by increasing sequencing depth.

A third approach to increase the number of SNPs discovered could be to use an enzyme or combination of enzymes that would provide a larger number of digested fragments and increasing the depth of sequencing. To investigate this, we extended the *in silico* analysis of the peach genome sequence to include consideration of two-enzyme combinations. The results (Table S3 in File S1) indicated that the combination of *Ape*KI and *Hpa*II might provide a substantially higher number of fragments of suitable length than *Ape*KI alone.

While GBS can generate large numbers of polymorphic markers, it can suffer from incorrect assignment of parental phase, underestimation of heterozygotes, and high proportions of missing data (Lu *et al.* 2013). Here, technical replicates of the parents were included in the genomic library and very stringent filters were applied to select subsets of markers and progeny for initial mapping. During map construction, diagnostic tests were conducted to detect and correct phasing errors. These approaches contributed to a very high success rate in KASP assay design. Only one incorrectly phased marker was detected. That marker (GBS tag pair TP37439), which had originally been assigned to the Nonpareil map, was reassigned to the Lauranne map based on results obtained with the WriPdK92 primer set. Three markers (GBS tag pairs TP15642, TP16449, and TP18643) that were originally assigned to the Lauranne map were determined to be heterozygous in both parents and were not used for map construction. Three other markers (GBS tag pairs TP11609, TP12109, and TP25403) that had originally been scored as heterozygous in one parent were determined to be homozygous in both parents and were not used for map construction. With the KASP markers, it was possible to obtain complete and accurate data for a larger number of progeny than had been used for the initial GBS map. Therefore it is not surprising that there are some differences in marker order between the initial and KASP maps; the KASP maps should be considered as more reliable.

The numbers of GBS tag pairs used to construct the initial Nonpareil and Lauranne linkage maps (310 and 282, respectively) were similar to numbers that have been used for Japanese plum (232 for one parent and 324 for the other; Salazar *et al.* 2017) and for sweet cherry (443 for one parent and 474 for the other; Guajardo *et al.* 2015). The initial genetic maps constructed using GBS data are about twice as long as the Nonpareil and Lauranne maps published by Tavassolian *et al.* (2010), but the final maps constructed using data from KASP assays are similar in length to the previously published maps. This “shrinkage” was due to correction of genotypes that had been erroneously called in the GBS analysis. In most cases, corrections were from homozygous to heterozygous, indicating that although two alleles were present, only one of them had been sequenced in sufficient depth. Of a total of 12,720 heterozygous calls in the final KASP data set, 1526 (12%) had been miscalled as homozygous in the GBS analysis. This type of error was evenly distributed among markers. These observations are similar to what has been reported for GBS analysis in switchgrass (Lu *et al.* 2013).

The success rate in converting SNP-bearing tag pairs to useful fluorescence-based marker assays was very high. Of the 309 SNP-bearing GBS sequences that were selected for assay design, 293 (95%) were successfully converted to KASP assays and mapped (138 for Nonpareil and 155 for Lauranne). However, not all of these could have been designed solely based on the GBS data. In many tags, the SNP position was too close to one end of the tag for primer design. These tag sequences had to be aligned against preexisting contig sequences to obtain flanking sequences.

Until now, linkage mapping in almond has relied on data from the progeny of individual biparental crosses. Here, progeny from four Nonpareil crosses were used, providing four linkage maps for Nonpareil. There should be no differences in the true biological positions of the SNPs on these four maps, as each map is based on estimates of recombination frequencies for the same parent, Nonpareil. While there was very good agreement among the maps, there were also some differences, presumably due to sampling error. Given that the SNPs mapped in all four populations had been preselected because they were informative for N × L, the map derived from that population has most markers and the best genome coverage. Among the four individual Nonpareil maps, the one from N × L should be considered as the most reliable. Nevertheless, the quality of any genetic map is limited by sample size. Here, the availability of additional Nonpareil populations provided an opportunity to improve the map accuracy and resolution. The Nonpareil composite linkage map constructed here is the first almond linkage map constructed based on genotypic data from multiple cross combinations. The use of four crosses with a common parent (Nonpareil) provided an opportunity to exploit a larger total population size to resolve the order of some closely linked markers. Given that Nonpareil is the predominant almond cultivar in both the United States and Australia, this linkage map should be a particularly useful new resource for almond research, including QTL mapping and marker-assisted breeding.

To demonstrate the utility of the linkage maps for QTL mapping and to provide examples of markers that could be used for selection, QTL results are presented here for shell hardness. For that trait, QTL were detected on three linkage groups: LG2, LG5, and LG8. QTL for this trait had previously been mapped on LG2 (Sánchez-Pérez *et al.* 2007) and LG8 (Arús *et al.* 1998), but not on LG5. At all of these QTL, Nonpareil-like marker genotypes were associated with softer shells. Depending on whether the paper-shell characteristic of Nonpareil is considered favorable or unfavorable (too soft), selection could be imposed for or against Nonpareil-like genotypes at six markers (two on LG2, three on LG5, and one on LG8). In the N × L population, this would have either fixed or eliminated the paper-shell trait.

Given that this map was constructed using sequence-based markers, it was possible to anchor it to the peach genome sequence assembly. It will be possible to anchor it to almond genome sequence assemblies as they become available and to connect it with other sequence-based linkage maps as they are developed. Thus, this composite map could provide a platform for unification of genetic and genomic resources for the almond research community.

This is the first report of genome-wide anchoring of almond genetic maps to the peach whole genome sequence assembly. The results confirmed the expected high similarity between the almond and peach genomes, with only a few of the mapped markers anchoring to unexpected positions. In most parts of the genome, marker positions on Nonpareil and Lauranne genetic maps were linearly related with the physical positions to which they were anchored in the peach genome. There were some regions of the peach genome for which few almond polymorphisms were discovered. Based the data generated here, it is not possible to distinguish whether these are simply regions in which Nonpareil and Lauranne are both highly homozygous, or whether these regions are structurally different between peach and almond.

The polymorphisms detected here among almond clones and rootstocks provide information about the transferability of SNPs discovered in one population to other materials. Of the markers designed based on heterozygosity in Nonpareil or Lauranne, 70% were useful for polymorphism detection among almond rootstocks. Although SNPs are generally used for polymorphism detection within species, many of the SNP markers developed here based on almond polymorphism detected polymorphisms in material that originated from peach, apricot, plum, cherry, and complex backgrounds. The markers developed here could be broadly useful for detecting genetic differences among accessions of *P. dulcis*, related species, and interspecific crosses. Applications of these markers could include assessment of cultivar verification, genetic diversity assessment, genetic mapping, and marker-assisted selection. From among the markers that were assayed here, 11 were selected based on their ability to differentiate among 15 almond clones and 10 were selected based on their ability to differentiate among 15 rootstocks.

This is the first report on the use of GBS in almond to discover SNPs and to generate linkage maps. The processes that were used here to select a restriction enzyme, conduct GBS data analysis, and design KASP markers could be applied more broadly for almond and other species.

## Supplementary Material

Supplemental material is available online at www.g3journal.org/lookup/suppl/doi:10.1534/g3.117.300376/-/DC1.
